# Genetic Diversity in Candidate Single-Nucleotide Polymorphisms Associated with Resistance in Honeybees in the Czech Republic Using the Novel SNaPshot Genotyping Panel

**DOI:** 10.3390/genes16030301

**Published:** 2025-03-01

**Authors:** Martin Šotek, Antonín Přidal, Tomáš Urban, Aleš Knoll

**Affiliations:** 1 Department of Animal Morphology, Physiology and Genetics, Faculty of AgriSciences, Mendel University in Brno, Zemědělská 1, 613 00 Brno, Czech Republic; xsotek@mendelu.cz; 2 Department of Animal Breeding, Faculty of AgriSciences, Mendel University in Brno, Zemědělská 1, 613 00 Brno, Czech Republic

**Keywords:** *Apis mellifera*, Varroa resistance, VSH, SMR, immune response, SNP, genetic diversity, hygienic behavior

## Abstract

**Background/Objectives**: The increasing pressure from pathogens and parasites on *Apis mellifera* populations is resulting in significant colony losses. It is desirable to identify resistance-associated single-nucleotide polymorphisms (SNPs) and their variability for the purpose of breeding resilient honeybee lines. This study examined the genetic diversity of 13 SNPs previously studied for associations with various resistance-providing traits, including six linked to Varroa-specific hygiene, five linked to suppressed mite reproduction, one linked to immune response, and one linked to chalkbrood resistance. **Methods**: Genotyping was performed using a novel SNaPshot genotyping panel designed for this study. The sample pool consisted of 308 honeybee samples in total, covering all 77 administrative districts of the Czech Republic. **Results**: All examined loci were polymorphic. The frequency of positive alleles in our population is medium to low, depending on the specific SNP. An analysis of genotype frequencies revealed that most loci exhibited the Hardy–Weinberg equilibrium. A comparison of the allele and genotype frequencies of the same locus between samples from hives and samples from flowers revealed no significant differences. The genetic diversity, as indicated by the heterozygosity values, ranged from 0.05 to 0.50. The fixation index (*F*) was, on average, close to zero, indicating minimal influence of inbreeding or non-random mating on the genetic structure of the analyzed samples. **Conclusions**: The obtained results provide further insights into the genetic variation of SNPs associated with the immune response and resistance to pathogens in honeybee populations in the Czech Republic. This research provides a valuable foundation for future studies of honeybee diversity and breeding.

## 1. Introduction

Infectious diseases and parasites are among the main causes of mortality and colony losses of the western honey bee, *A. m.* Linnaeus, 1758. Their pressure on honeybee populations has increased in recent years due to the negative impact of environmental and anthropogenic factors (such as climate change and pesticide use) on bee health and the introduction of pathogens and parasites that local vulnerable bee populations were not adapted to [1,2,3,4]. This worsening trend could have a severe negative impact on global agriculture and food security in the upcoming years, as bees are crucial for pollination of agricultural crops [5,6]. Selective breeding for enhanced resilience could offer a potential solution, with genomic methods enabling the identification and selection of molecular markers associated with resistance mechanisms [7,8].

Honeybees have evolved diverse defense mechanisms against pathogens and parasites, which are involved at both individual and colony levels (social immunity). These include immune response and behavioral/physiological mechanisms such as grooming, hygienic behavior, and suppressed mite reproduction [9,10,11,12]. Analogous to human genetics [13], genetic polymorphisms in honeybees were found to be linked to pathogen and parasite resistance by influencing these defense responses. These polymorphisms may directly affect the phenotype or can be linked to causative mutations. Their occurrence and frequency are the subject of research in both susceptible and known resistant honeybee populations, as detailed below.

The immune system of honeybees is innate and consists of humoral and cellular innate responses [14,15]. Genetic differences in immune system components influence responses toward specific pathogens [16,17,18]. To assess immune gene diversity, Henriques et al. [19] designed a genotyping assay for screening 91 functional SNPs in 89 genes related to immune function. These polymorphisms were selected from a total number of 35,782 SNPs located in 180 immune genes using WGS data from 123 whole genomes from honeybee subspecies across three main lineages.

*Varroa destructor* Anderson and Trueman, 2000 represents one of the biggest threats to honeybee health, both on an individual and colony level [20,21]. Mites of this species weaken bees by feeding on their fat body cells and are possibly able to transmit viruses such as deformed wing virus (DWV), sacbrood virus (SBV), chronic bee paralysis virus (CBPV), and black queen cell virus (BQCV) [22,23,24,25]. This synergistic effect explains the mite’s devastating impact. Thus, breeding for Varroa resistance could, therefore, mitigate viral disease incidence [26,27].

The main defense mechanisms against *Varroa* mites that bees can employ are grooming behavior, Varroa-sensitive hygiene (VSH), and suppressed mite reproduction (SMR) [28]. By performing grooming behavior, adult bees remove parasites from themselves or other bees [29]. VSH, a form of hygienic behavior, involves workers detecting and removing mite-infested broods specifically [30,31]. Highly hygienic bees may exhibit increased sensitivity to stress molecules secreted by broods [32,33,34], suggesting genetic changes involving genes related to the function of the nervous system and antenna [35,36,37]. SMR involves the reduction or inhibition of *Varroa* reproduction through a brood-related trait, potentially involving changes in brood hormones and pheromones that regulate brood development [38,39,40,41]. This alteration disrupts the reproduction cycle of the founder mother mite, which is dependent on levels of brood chemicals ingested during feeding [39,42,43]. It can be further classified as drone-based reduction (DBR) and worker-based reduction (WBR) [42].

Several candidate polymorphisms associated with hygienic behavior and SMR have been identified through whole-genome sequencing (WGS), whole-exome sequencing (WES), genome-wide association study (GWAS), and gene expression analyses. Haddad et al. [44] and Texeira et al. [45] found SNP variants in genes associated with Varroa resistance in the genomes of the Syrian honey bee (*A. m. syriaca* Skorikov 1929) and Africanized bees, respectively. These two distinct subspecies are distinguished by their notably elevated levels of hygienic behavior, a trait that distinguishes them from European lineages, as previously documented by Carneiro et al. [46] and Kence et al. [47]. In experimental inbred lines, Kim et al. [48,49] identified autosomal (20) and mitochondrial (23) SNPs with variants specific to low VSH and high VSH workers of *A. m. carnica* Pollmann 1879 from Germany. Spötter et al. [50] identified six SNPs highly associated with this trait. Polymorphisms linked with SMR were described by Conlon et al. [43] and Broeckx et al. [42]. The former study found three non-synonymous SNPs in the *mblk-1* gene by studying a Varroa-resistant colony from Toulouse. *Mblk-1* is located on chromosome 15, with its expression controlled by ecdysone and its product functions being one of the metamorphosis regulators in larvae that is thought to influence *Varroa* mite reproduction [51]. In a comparative WES study, Broeckx et al. [42] selected the eight most significant SNPs with variant alleles associated with SMR traits in hybrid *A. mellifera* colonies in the Netherlands. Using a qPCR genotyping assay developed by Boúúaert et al. [52], Lefebre et al. [53,54] were able to screen for allele frequency of these polymorphisms in six subspecies across 14 European countries and to confirm and evaluate their association with the trait in *A. m. carnica* Pollman 1879 colonies (Flanders) by population-wide modeling, producing two updated association models.

Chalkbrood disease, also known as ascosphaerosis, is an invasive mycosis that affects the bee brood. It is caused by the cosmopolitan fungus *Ascosphaera apis* (Maasen ex Claussen) L.S. Olive and Spiltoir, 1955. While not directly causing colony collapse, it has been observed to weaken colonies and reduce honey yields [55,56]. Holloway et al. [57,58] found several candidate polymorphisms related to chalkbrood resistance on QTLs located on chromosomes 2 and 11. In *A. m. ligustica* colonies from the Zhejiang and Fujian provinces in China, Liu et al. [59] identified three candidate SNPs in a second intron of *major royal jelly protein 5* (*mrjp5*) gene on chromosome 11 (in a different region compared to Holloway et al.) [58], with one of them being significantly associated with chalkbrood resistance.

For this study, we selected 13 candidate SNP markers (Table 1) associated with immune response, Varroa resistance, and chalkbrood resistance in honeybees. From six VSH-linked SNPs identified by Spötter et al. [50] using 44K SNP array, SNP1 (described as AMB-00457689) and SNP2 (described as AMB-00745078) were selected. According to NCBI Genebank, SNP1 is in the *E23* gene, which appears to be orthologous to the ABC transporter in *Drosophila melanogaster*, whose product has a possible function in regulating circadian rhythms [59], whereas SNP2 lies in still unannotated lncRNA locus LOC113218862.

Four of the selected polymorphisms—SNP3, SNP4, SNP5, and SNP6—were among those identified by Haddad et al. [44] as present in more resilient *A. m. syriaca* by alignment of WGS sequences with reference sequences. They are located within genes presumably linked to Varroa-resistant traits (mainly VSH) that play a role in the development, function, and signaling of the nervous system (ankyrin—membrane–cytoskeleton interaction; futsch—microtubule-associated protein; Arms—scaffold protein; Kr-h—homologous to kruppel homeotic gene/transcription factor).

SMR-associated markers—SNP7, SNP8, SNP9, SNP10, and SNP11—are part of the original eight-variant model identifying six risk and two protective alleles developed by Broeckx et al. [42] as a part of a WES study on low and high SMR/DBR pupae from hybrid *A. m. mellifera* colonies in the Netherlands. Lefebre et al. [54] developed two improved models for the *A. m. carnica* subspecies—an eight-variant model and a reduced three-variant model, including three statistically significant polymorphisms. SMR-linked SNPs are located in genes that have possible involvement in olfactory neurons of antenna and regulation of production and transport of brood pheromones, which are mechanisms that were previously hypothesized to be linked to SMR trait.

The potential immune response marker SNP12 was identified by Henriques et al. [19] for a medium-density genotyping assay containing 91 quality-proven functional SNPs and was described as HB186. It lies within the gene for C-type lectin 2, which functions as a pattern recognition receptor (PRR) in the immune system [14,60]. This SNP could be used as a marker for genetic variability of immune genes, with observed differential expression in bees infected with the Israeli acute paralysis virus IAPV [61].

**Table 1:** Candidate single-nucleotide polymorphisms (SNPs) selected for SNaPshot assay.

SNP	Gene (Gene Symbol) *	Associated Trait	Alleles (+Strand)	Positive Allele **	Type	RefSNP	Genome Position (Amel_HAv3.1)	Source
SNP1	GB49098, *Early gene at 23 (E23)*	VSH	T/C	C	5′UTR	rs44529083	LG3: 3646748	[50]
SNP2	*Uncharacterized LOC113218862 (LOC113218862)*	VSH	T/C	C	lncRNA locus	unassigned	LG6: 2816682	[50]
SNP3	GB46564, *Ankyrin-3 (LOC409051)*	VR/VSH	T/C	C	Synonymous	rs45199156	LG4: 9970541	[44]
SNP4	GB49223, *microtubule-associated protein futsch (LOC725131)*	VR/VSH	C/T	T	Synonymous	rs883050117	LG7: 3779983	[44]
SNP5	GB51019, *kinase D-interacting substrate of 220 kDa/Ankyrin repeat-rich membrane spanning (Arms)*	VR/VSH	G/A	A	Synonymous	rs882879128	LG14: 5507078	[44]
SNP6	GB45427, *kruppel homolog 1 (Kr-h1)*	VR/VSH	A/G	G	Synonymous	rs45205634	LG6: 16506998	[44]
SNP7	GB53340, *spectrin beta chain isoform X1 (beta-Spec)*	SMR	T/G	T (G is a risk allele)	Synonymous	rs7016398622	LG9: 11461121	[42,53,54]
SNP8	GB53345, *uncharacterized protein LOC100578770 (LOC100578770)*	SMR	A/G	A (G is a risk allele)	Non-synonymous (Met/Val)	rs882600476	LG9: 11542136	[42,53,54]
SNP9	GB17622, *Mucin-12 isoform X1 (LOC412088)*	SMR	A/G	G (protective allele)	Synonymous	rs884078745	LG1: 24214744	[42,53,54]
SNP10	GB48382, *solute carrier family 22 member 21 (LOC408302)*	SMR	G/A	G (A is a risk allele)	Synonymous	rs7017327617	LG10: 6509050	[42,53,54]
SNP11	GB11211, GB47018, *uncharacterized protein LOC724886 (LOC724886)*	SMR	C/T	C (T is a risk allele)	Synonymous	rs882593377	LG3: 11807235	[42,53,54]
SNP12	GB45248, *C-type lectin 2/CTL2 (LOC410154)*	Immune response	A/G	Not assessed	Non-synonymous (Val/Lys)	rs881621875	LG11: 14820214	[19]
SNP13	GB55208, GB10622, *major royal jelly protein 5 (Mrjp5)*	Chalkbrood resistance	C/T	C	Intron	rs882017294	LG11: 2292265	[62]

* Gene designation according to NCBI database. VR: Varroa resistance; VSH: Varroa-specific hygiene; SMR: suppressed mite reproduction. ** Positive alleles are linked to higher resistance.

SNP13 in the second intron of the *mrjp5* gene was found to be associated (based on a comparison of the frequency of the C allele) with chalkbrood resistance [62]. MRJPs are important for the nutrition and development of larvae, and some of them have confirmed antimicrobial activity; however, the potential mechanism of *mrjp5*-mediated chalkbrood resistance remains unknown [63,64].

The aim of this study is to determine the diversity of 13 honeybee resistance SNPs in the Czech honeybee population. This study screens allele and genotype frequencies, as well as diversity parameters of candidate polymorphisms in honeybees sampled from colonies and individual workers caught on flowers across the country. The results could be useful for future breeding efforts using molecular markers to obtain resistant populations and to understand the genetic variability of honeybee populations.

## 2. Materials and Methods

### 2.1. Sampling of Bees

The Czech Republic is comprised of 77 districts, including the capital city of Prague. The average size of a district is 1024 km^2^. Two types of sampling were conducted: in hives and on flowers. The two hive samples were taken from colonies in different parts of each district and from different beekeepers. The flower samples were obtained by catching free-flying bees at two mutually remote locations per district. The flower samples were collected at a sufficient distance from the nearest sampled apiary to avoid duplication. In 2022 and 2023, a total of 308 samples were obtained. After the sampling, the material was stored at −20 °C until the DNA isolation.

### 2.2. DNA Extraction

Genomic DNA was extracted using a standard protocol (Tissue Genomic DNA Mini Kit, Geneaid, Taipei, Taiwan). Genomic DNA was extracted from thoracic muscle tissues.

### 2.3. SNP Selection

A novel SNaPshot assay was developed and used for genotyping of candidate polymorphisms in samples obtained from beekeepers from across the Czech Republic. Candidate SNPs for the assay were selected from a set of 32 polymorphisms in 29 genes. Test samples (*n* = 20) were then genotyped for these SNPs using Sanger sequencing. The main criteria for selection were allele variability in the test sample set. As a result, only the 13 markers were polymorphic and included in the assay; the other 19 markers were monomorphic or with a low frequency (<5%) of the minor allele. The SNP markers that were not included in the SNaPshot panel are listed in Supplementary Table S1a,b.

### 2.4. Multiplex PCR Amplification

For SNaPshot genotyping assay, we selected two random hive samples and two random flower samples from each district in our genetic bank. First, we designed optimal PCR primers from flanking sequences (300 bp on both sides) of each SNP. The primers were designed using Primer Express v2.0 software, considering the same melting temperature required for multiplexing. These primers were used for both Sanger sequencing and for subsequent multiplex PCR for SNaPshot assay. Designed PCR primers are listed in Table S2. PCR products in singleplex were verified by agarose electrophoresis and by Sanger sequencing. No non-specific products were detected that could negatively impact the quality of the results. Microchip electrophoresis (Shimadzu MCE-202 MultiNA, DNA-500 kit, Shimadzu Corporation, Kyoto, Japan) was used for multiplex PCR validation. For the assay, we subsequently created and optimized three multiplexes—MP1 (SNP2, SNP3, SNP4, SNP6, and SNP13), MP2 (SNP1, SNP5, SNP8, and SNP9), and MP3 (SNP7, SNP10, SNP11, and SNP12). All reactions were carried out in a total volume of 10 µL, using 0.5 µL of template DNA and 1× Combi PPP Master Mix (Top-Bio, Vestec, Czech Republic), which contained 0.5 U of HotStar Taq polymerase, 200 µM of total dNTP, and 2.5 mM of MgCl_2_. Detailed information on PCR mix composition for each multiplex, including primer volume, is listed in Table S3a–c. The cycling conditions were as follows: 95 °C (2 min), 30 cycles of a 30 s denaturation at 95 °C, a 30 s annealing at 58 °C, a 45 s elongation at 72 °C, and final extension step at 72 °C for 10 min. PCR was performed using an AB Veriti Dx 96-well thermocycler (Applied Biosystems, Foster City, CA, USA).

### 2.5. PCR Product Pooling and Purification

Before the one-base extension step, we pooled the three amplicon multiplexes for each sample in one 6 µL (2 µL of each MP) mix containing all 13 amplicons and added 2.0 µL of Shrimp Alkaline Phosphatase (SAP, 1 U/µL, Thermo Fischer Scientific Baltics UAB, Vilnius, Lithuania) and 0.1 µL of Exonuclease 1 (EXO1, 10 U/µL, Thermo Fischer Scientific Baltics UAB, Vilnius, Lithuania). The mixture containing enzymes was then incubated for 1 h at 37 °C, followed by 15 min at 75 °C for removal of unincorporated primers and dNTPs and subsequent enzyme deactivation.

### 2.6. Extension and Post-Extension Treatment

SNaPshot method is based on dideoxynucleotide single-base extension at the SNP site. For one-base extension at SNP site, we designed specific extension primers for each of the 13 SNPs. Primers (forward or reverse) were designed to anneal close to the SNP, with the 3′ end positioned one nucleotide upstream of the SNP. To differentiate primers on the same strand by length, a variable number of GACT repeats were added to the 5′ end. SNaPshot extension primers are listed in Table S2. Reaction mixture for extension contains 1.25 µL of SNaPshot™ Multiplex Kit (Life Technologies LTD, Warrington, UK), 2.2 µL of Big Dye Terminator Sequencing buffer 5× (Thermo Fischer Scientific Baltics UAB, Vilnius, Lithuania), 0.2 µL of SNaPshot primers (each), 1 µL of deionized water, and 3 µL of pooled and purified PCR products. The extension reaction was performed using AB Veriti thermocycler with the following conditions: 25 cycles of a 10 s denaturation at 96 °C, 5 s annealing at 55 °C, and 30 s extension at 60 °C. Post-extension purification was performed by adding 0.5 µL of SAP to 5 µL of extension product, followed by the same incubation as the post-PCR purification.

### 2.7. Genotyping

Capillary electrophoresis was performed on ABI PRISM 3500 Genetic Analyser (Applied Biosystems, Foster City, CA, USA) using standard conditions (POP-7 polymer, G5 matrix) and the SNaPshot analyzing protocol. Fragment size was accurately determined using GeneScan™ 120 LIZ™ Size Standard, and genotypes were determined using GeneMapper v 6.0 software (Applied Biosystems, Foster City, CA, USA). An example of genotype evaluation based on electrophoresis output is given in Figure S1. The validity of the developed methodology was verified by Sanger sequencing of all 13 SNPs in 39 randomly selected samples.

### 2.8. Data Analysis

Using GenAlEx v6.5 [65], we performed genetic analyses on hive and flower samples, calculating allele and genotype frequencies. Deviations from Hardy–Weinberg equilibrium (HWE) were assessed using an exact test. Genetic diversity within each sample was quantified by calculating the number of alleles (*Na*), effective number of alleles (*Ne*), Shannon’s information index (*I*), observed heterozygosity (*Ho*), expected heterozygosity (*He*), unbiased expected heterozygosity (*uHe*), and the fixation index (*F*), which measures of inbreeding within a population, indicating the degree of non-random mating and genetic structure.

Fisher’s exact tests were used to determine the statistical significance of differences in both allelic and genotypic frequencies between hive and flower samples. Paired *t*-tests were used to compare genetic diversity parameters between the two sample types. All analyses were conducted in R, version 4.4.2 [66].

## 3. Results

### 3.1. SNAPShot Evaluation

The validity of the developed SNaPshot methodology was verified by Sanger sequencing of all 13 SNPs in 39 randomly selected samples. Obtained sequences of all found alleles are presented in File S1.

### 3.2. Genetic Diversity

Allele frequencies at analyzed SNP loci in hive and flower bee samples are presented in Table 2. All loci were polymorphic in both samples. Minor differences in allele frequencies were observed between the two groups, but no major shifts or fixed differences were detected. A comparison of allele frequencies in honeybees sampled from hives and while foraging on flowers revealed both similarities and differences across the analyzed loci. Allele frequencies at several loci (SNP1, SNP5, SNP8, SNP9, and SNP11) showed minimal variation between the two sample groups. SNP5 was nearly monomorphic for the G allele. The other loci showed higher differences in allele frequencies. SNP2 and SNP3 showed similar patterns: the T allele was dominant in both hive and flower samples, but its frequency was slightly lower in flower samples (0.851 and 0.834, respectively) than in hive samples (0.893 and 0.867, respectively). In contrast, SNP4, SNP7, and SNP13 showed more pronounced differences. The T allele frequency increased in flower samples compared to hive samples for SNP4 (0.240 vs. 0.185) and SNP7 (0.588 vs. 0.513). A similar pattern was observed for SNP13, with the C allele also showing an increased frequency in flower samples (0.292 vs. 0.211). Finally, SNP6, SNP10, and SNP12 exhibited slightly higher A allele frequencies in flower samples than in hive samples. Statistically significant differences (*p* < 0.05) in allele frequencies were found only for SNP12 and SNP13 between hive and flower samples.

The analysis demonstrates considerable variation among loci in the frequency of alleles associated with resistance. Most loci, specifically those associated with VSH, VR, immune response, and chalkbrood resistance, showed low frequencies of the positive alleles (SNP1–C, SNP2–C, SNP3–C, SNP4–T, SNP5–A, SNP6–G, SNP12–G, and SNP13–C). In contrast, the SMR-associated loci displayed moderate allele frequencies (SNP7–T, SNP8–G, SNP9–G, SNP10–G, SNP11–C).

Table 3 presents genotype frequencies and Hardy–Weinberg equilibrium (HWE) *p*-values for SNP loci in hive and flower samples. Most loci conformed to HWE in both sample types, with generally consistent genotype frequencies between hives and flowers. However, two loci (SNP5 and SNP12) showed significant HWE deviations in hive samples due to extremely low frequencies of the AA and GG genotypes, respectively. SNP10 also showed a significant HWE deviation, but only in the flower samples.

Analysis of genotype frequencies revealed distinct patterns of allelic variation among loci. The high prevalence of the TT genotype at SNP1, SNP2, SNP3, and SNP13 indicates a correspondingly high frequency of the T allele. Conversely, the prevalence of CC or AA homozygotes at SNP4, SNP6, and SNP12 reflects increased C and A allele frequencies. The SNP5 locus was nearly fixed for the GG genotype, indicating near fixation of the G allele. In contrast to these loci with a higher proportion of homozygotes, SNP7, SNP8, SNP9, SNP10, and SNP11 exhibited a more balanced distribution of genotypes characterized by elevated heterozygosity. This suggests either a lack of strong directional selection or the operation of balancing selection mechanisms. Statistically significant differences in genotype frequencies between hive and flower samples were found for SNPs SNP12 (*p* < 0.01) and SNP13 (*p* < 0.05).

Analysis of genetic diversity parameters (effective number of alleles [*Ne*], Shannon’s index [*I*], observed [*Ho*] and expected [*He*] heterozygosity, and unbiased expected heterozygosity [*uHe*]) in honeybees sampled from hives reveals interesting findings about variability among individual loci (in Table 4). Loci were grouped into three categories based on their genetic diversity parameters. High diversity was observed at SNP8, SNP9, and SNP11 (*Ne* ≈ 2, I ≈ 0.69, *Ho* > 0.53, *He* ≈ 0.5, *uHe* ≈ 0.5), indicating a balanced allelic ratio and high genetic variability. Moderate diversity was observed at SNP1, SNP3, SNP4, SNP7, SNP10, and SNP13 (*Ne*: 1.3–1.8, *I*: 0.39–0.64, *Ho*: 0.25–0.47, *He* and *uHe*: 0.23–0.45). Low diversity was found at SNP2, SNP5, SNP6, and SNP12 (*Ne*: 1.05–1.34, *I*: 0.12–0.42, *Ho*: 0.039–0.299, *He* and *uHe*: 0.051–0.255), with SNP5 exhibiting particularly low diversity. The fixation index (*F*) varied among loci. Negative *F* values, indicative of a slight excess of heterozygotes relative to Hardy–Weinberg equilibrium, were observed at most loci, while positive *F* values, suggesting a slight deficit, were found at some (SNP5, SNP7). The near-zero mean *F* value across all loci suggests that inbreeding has a minimal overall effect on the genetic structure of the analyzed samples.

The analysis of genetic diversity in honeybees collected from flowers (Table 5) reveals similar patterns to those observed in hive samples. Observed (*Ho*) and expected (*He*) heterozygosity are generally low to moderate across all loci, 0.342 and 0.455, respectively. The mean fixation index (*F*) is close to zero (0.009). The high-diversity group (SNP7, SNP8, SNP9, and SNP11) exhibited *Ne* values approaching 2, *I* values ranging from 0.68 to 0.69, *Ho* values ranging from 0.42 to 0.53, and *He* values ranging from 0.48 to 0.50, consistent with balanced allele frequencies. The moderate-diversity group (SNP1, SNP3, SNP4, SNP10, and SNP13) showed intermediate values for these parameters: *Ne* (1.38–1.74), *I* (0.45–0.62), *Ho* (0.27–0.44), and *He* (0.28–0.43). The low-diversity group (SNP2, SNP5, SNP6, and SNP12) exhibited lower values: *Ne* (1.05–1.34), *I* (0.12–0.42), *Ho* (0.05–0.22), and *He* (0.05–0.25). Among these, SNP5 consistently displayed the lowest diversity. With the exception of SNP10, most loci exhibited negative *F* values, indicating a slight excess of heterozygotes. SNP10 showed a small positive *F* value (0.163), suggesting a slight deficiency of heterozygotes. A *t*-test revealed no statistically significant difference in all diversity parameters between the two sample groups (*p* > 0.05).

## 4. Discussion

Previous research has demonstrated the presence of SNPs linked to increased resistance in managed honeybee populations not specifically bred for resistance [42,53]. We present data on the occurrence and genetic diversity of 13 candidate SNP markers potentially associated with immune response, Varroa resistance, and chalkbrood resistance in honeybees. The SNaPshot method was chosen for genotyping due to its proven efficiency and cost-effectiveness for a wide range of applications [67,68,69]. Compared to alternative methods such as Sanger sequencing, we have found it to be inexpensive, fast, and reliable for routine testing of multiple SNPs.

A genetic analysis of honeybees collected from hives and flowers, based on biallelic SNPs (n = 308), revealed similar overall diversity patterns across samples. In general, allele frequencies were comparable between hive and flower samples, indicating a representative population. Nevertheless, the observed differences at specific loci, notably SNP12 and SNP13, may be due to random sampling despite the similar sample sizes.

Most loci (SNP1, SNP2, SNP3, SNP4, SNP6, SNP7, SNP8, SNP9, SNP11, and SNP13) showed no significant deviations from HWE in either sample, indicating random mating within both hive and flower samples for these loci. Significant deviations observed at SNP5 (in hives), SNP10 (on flowers), and SNP12 (in hives) suggest the influence of evolutionary forces, potentially including locus-specific selection, non-random mating, or population structure. Furthermore, balancing selection or heterozygote advantage may maintain polymorphisms at certain SNPs, while directional or purifying selection could reduce genetic diversity at others [70]. While genotype distributions were generally similar across sample sources, some SNPs exhibited moderate variations.

Loci were categorized according to their level of genetic diversity: high, moderate, or low. The high diversity observed at SNP8, SNP9, and SNP11 suggests that these loci may be under balanced selection. In contrast, loci with moderate or low diversity may be influenced by different selective pressures. Notably, SNP2, SNP5, SNP6, and SNP12 demonstrated reduced genetic diversity, with SNP5 consistently displaying the lowest diversity among all loci examined.

The negative *F* values at most loci (though close to zero) suggest a tendency towards outbreeding, consistent with the known polyandrous mating behavior of honeybees. The higher positive *F* values observed at SNP5 and SNP7 warrant further investigation to determine the underlying causes. While individual loci show some deviation from Hardy–Weinberg equilibrium, the near-zero mean *F* value suggests that inbreeding is not a major factor shaping the population’s overall genetic structure. This near-zero *F* value further reflects the reproductive strategy of honeybees and the absence of strong selection pressure for increased resistance due to rearing interventions, indicating no significant deviations in heterozygosity from HWE.

The Varroa-specific hygienic behavior markers SNP1 and SNP2 exhibited relatively low frequencies of positive alleles (SNP1–C: 0.286/0.263; SNP2–C: 0.107/0.149) in hives and flowers samples, respectively. This suggests that these alleles are not under strong positive selection, indicating minimal numbers of homozygous individuals. SNP3, SNP4, SNP5, and SNP6 previously identified in *A. m. syriaca* as being located in genes associated with varroosis resistance and hygienic behavior [44], also showed low frequencies of their positive variants in this study (SNP3–C: 0.133/0.166; SNP4–T: 0.185/0.240; SNP5–A: 0.026/0.026; SNP6–G: 0.114/0.094). The A allele at SNP5 was observed in a homozygous state in only one sample. The lower frequencies of positive alleles at SNPs 3, 4, 5, and 6, compared to SNPs 1 and 2, suggest a possible link between these allele frequencies and reduced VSH in the Czech population.

SNP7, SNP8, SNP9, SNP10, and SNP11 were selected for their association with SMR, specifically drone-based reduction (DBR) [42,54]. We prioritized the models developed by Lefebre et al. [54] because they studied *A. m. carnica*, the subspecies most closely matching Czech Republic populations [71], and used a larger study population. The variant allele was risky at SNPs 7, 8, 10, and 11 (positive alleles are wild-type) and protective at SNP 9 [54]. Variant allele frequencies in European *A. m. carnica* [52] and in our hive and flower samples were as follows: SNP7–G: 0.520/0.487/0.412; SNP8–G: 0.597/0.558/0.555; SNP9–G: 0.645/0.513/0.519, SNP10–A: 0.521/0.659/0.692; and SNP11–T: 0.441/0.481/0.477. While allele frequencies at the SMR markers exhibited some variability, they were generally comparable. These markers were characterized by a relatively high representation of both allelic variants and a high degree of heterozygosity.

SNP12, located in the CTL2 immune gene, exhibits a high frequency of the A allele (0.851/0.912) and a low frequency of the G allele (0.149/0.088). Most samples carrying the G allele were heterozygous; only one of the 308 samples was homozygous. This deviation from the Hardy–Weinberg equilibrium may be due to selection pressure, non-random mating, or the Wahlund effect. Further research is needed to determine the relationship between these SNP12 variants and immune response and to verify their association with IAPV defense.

SNP13 is located within the second intron of the *Mrjp5* gene and has been associated with chalkbrood resistance based on a comparative analysis of allele frequencies between individuals from chalkbrood-resistant and chalkbrood-susceptible colonies [59]. In our study, the C allele frequency was 0.211 in hive samples and 0.292 in flower samples. In contrast, Liu et al. [59] reported C allele frequencies of 0.660, 0.489, and 0.614 in three resistant colonies and 0.225, 0.351, and 0.457 in three susceptible colonies. The frequencies observed in our hive and flower samples are more similar to those of susceptible colonies reported by Liu et al., suggesting that our study population is likely to have a low level of resistance to chalkbrood.

The results of this study on the current variability of the studied SNPs should be applied in follow-up studies investigating their linkage with phenotypes in a selectively bred strain developed using artificial insemination, with long-term selection for desirable vigor-related traits. An alternative experimental approach would involve applying artificial insemination to create a line enriched for positive alleles and subsequently testing its phenotype. The first approach requires a long-term selectively bred population, while the second relies on a highly variable allelic population.

## 5. Conclusions

This study is the first to assess the diversity of resistance-related SNP markers in Czech honeybee populations. A SNaPShot assay was designed and tested for 13 selected single-nucleotide polymorphisms presumably involved in disease and parasite resistance. The results obtained revealed variability in these SNPs in the *A. m. carnica* population in the Czech Republic. The genetic diversity profiles of honeybees sampled from hives and flowers were largely similar, particularly in relative diversity levels across loci and the near-zero mean fixation index. Beneficial variants of Varroa-specific hygiene and chalkbrood resistance-linked SNPs were present but at low frequencies, suggesting potential vulnerability to these pressures. High frequencies of positive alleles and heterozygosity at SMR-associated loci made it difficult to assess this trait in our population. Knowledge of this variability in the honeybee population in the Czech Republic may serve as a valuable resource for future research aimed at establishing associations between genotypes and relevant phenotypic traits.

## Figures and Tables

**Table 2:** Allele frequencies of SNPs in *A. m.* collected from hives and flowers.

		Allele Frequency	
Locus	Allele	Hives	Flowers
SNP1	C	0.286	0.263
	T	0.714	0.737
SNP2	C	0.107	0.149
	T	0.893	0.851
SNP3	C	0.133	0.166
	T	0.867	0.834
SNP4	C	0.815	0.760
	T	0.185	0.240
SNP5	A	0.026	0.026
	G	0.974	0.974
SNP6	A	0.886	0.906
	G	0.114	0.094
SNP7	G	0.487	0.412
	T	0.513	0.588
SNP8	A	0.442	0.445
	G	0.558	0.555
SNP9	A	0.487	0.481
	G	0.513	0.519
SNP10	A	0.659	0.692
	G	0.341	0.308
SNP11	C	0.519	0.523
	T	0.481	0.477
SNP12	A	0.851	0.912
	G	0.149	0.088
SNP13	C	0.211	0.292
	T	0.789	0.708

Alleles and their frequencies marked in blue are positive alleles for resistance if designed.

**Table 3:** Genotype frequencies of SNPs and the genetic equilibrium (HWE).

	Sampled in Hives				Sampled on Flowers			
Locus	Genotype Frequency			HWE (p)	Genotype Frequency			HWE (p)
SNP1	CC	CT	TT	0.338 ^ns^	CC	CT	TT	0.492 ^ns^
	0.0974	0.3766	0.5260		0.0584	0.4091	0.5325	
SNP2	CC	CT	TT	0.299 ^ns^	CC	CT	TT	0.104 ^ns^
	0.0195	0.1753	0.8052		0.0390	0.2208	0.7403	
SNP3	CC	CT	TT	0.227 ^ns^	CC	CT	TT	0.650 ^ns^
	0.0065	0.2532	0.7403		0.0325	0.2662	0.7013	
SNP4	CC	CT	TT	0.496 ^ns^	CC	CT	TT	0.624 ^ns^
	0.6558	0.3182	0.0260		0.5844	0.3506	0.0649	
SNP5	AA	GA	GG	0.004 **	AA	GA	GG	0.741 ^ns^
	0.0065	0.0390	0.9545		-	0.0519	0.9481	
SNP6	AA	GA	GG	0.993 ^ns^	AA	GA	GG	0.730 ^ns^
	0.7857	0.2013	0.0130		0.8182	0.1753	0.0065	
SNP7	GG	GT	TT	0.149 ^ns^	GG	GT	TT	0.109 ^ns^
	0.2662	0.4416	0.2922		0.2013	0.4221	0.3766	
SNP8	AA	GA	GG	0.323 ^ns^	AA	GA	GG	0.258 ^ns^
	0.1753	0.5325	0.2922		0.1753	0.5390	0.2857	
SNP9	AA	GA	GG	0.255 ^ns^	AA	GA	GG	0.409 ^ns^
	0.2143	0.5455	0.2403		0.2143	0.5325	0.2532	
SNP10	AA	GA	GG	0.496 ^ns^	AA	GA	GG	0.043^*^
	0.4221	0.4740	0.1039		0.5130	0.3571	0.1299	
SNP11	CC	CT	TT	0.409 ^ns^	CC	CT	TT	0.727 ^ns^
	0.2532	0.5325	0.2143		0.2662	0.5130	0.2208	
SNP12	AA	GA	GG	0.029 *	AA	GA	GG	0.853 ^ns^
	0.7013	0.2987	-		0.8312	0.1623	0.0065	
SNP13	CC	CT	TT	0.300 ^ns^	CC	CT	TT	0.402 ^ns^
	0.0584	0.3052	0.6364		0.0714	0.4416	0.4870	

Chi-square tests for Hardy–Weinberg equilibrium: ns = not significant; * *p* < 0.05; ** *p* < 0.01.

**Table 4:** Parameters of the genetic diversity of *A. m.* collected from hives.

Locus	*n*	*Na*	*Ne*	*I*	*Ho*	*He*	*uHe*	*F*
SNP1	154	2.000	1.690	0.598	0.377	0.408	0.409	0.077
SNP2	154	2.000	1.237	0.340	0.175	0.191	0.192	0.084
SNP3	154	2.000	1.300	0.392	0.253	0.231	0.232	−0.097
SNP4	154	2.000	1.432	0.479	0.318	0.302	0.303	−0.055
SNP5	154	2.000	1.053	0.120	0.039	0.051	0.051	0.230
SNP6	154	2.000	1.252	0.354	0.201	0.201	0.202	0.001
SNP7	154	2.000	1.999	0.693	0.442	0.500	0.501	0.116
SNP8	154	2.000	1.973	0.686	0.532	0.493	0.495	−0.080
SNP9	154	2.000	1.999	0.693	0.545	0.500	0.501	−0.092
SNP10	154	2.000	1.816	0.642	0.474	0.449	0.451	−0.055
SNP11	154	2.000	1.997	0.692	0.532	0.499	0.501	−0.067
SNP12	154	2.000	1.341	0.422	0.299	0.254	0.255	−0.176
SNP13	154	2.000	1.499	0.515	0.305	0.333	0.334	0.084
Mean	154.000	2.000	1.584	0.510	0.346	0.339	0.341	−0.002
SE	0.000	0.000	0.095	0.050	0.043	0.041	0.041	0.031

*n*, number of samples; *Na*, No. of different alleles; *Ne*, No. of effective alleles = 1/(Σp_i_^2^); *I*, Shannon’s information index = −1 × Σ(p_i_ × Ln(p_i_)); *Ho*, observed heterozygosity = No. of Hets/*N*; *He*, expected heterozygosity = 1 − Σp_i_^2^; *uHe*, unbiased expected heterozygosity = (2*N*/(2*N* − 1)) × *He*; *F*, fixation index = (*He* − *Ho*)/*He* = 1 − (*Ho*/*He*); p_i_ is the frequency of the i^th^ allele for the population.

**Table 5:** Parameters of the genetic diversity of *A. m.* collected from flowers.

Locus	*n*	*Na*	*Ne*	*I*	*Ho*	*He*	*uHe*	*F*
SNP1	154	2.000	1.633	0.576	0.409	0.388	0.389	−0.055
SNP2	154	2.000	1.341	0.422	0.221	0.254	0.255	0.131
SNP3	154	2.000	1.382	0.449	0.266	0.276	0.277	0.037
SNP4	154	2.000	1.575	0.551	0.351	0.365	0.366	0.040
SNP5	154	2.000	1.053	0.120	0.052	0.051	0.051	−0.027
SNP6	154	2.000	1.206	0.312	0.175	0.171	0.171	−0.028
SNP7	154	2.000	1.940	0.678	0.422	0.485	0.486	0.129
SNP8	154	2.000	1.976	0.687	0.539	0.494	0.496	−0.091
SNP9	154	2.000	1.997	0.692	0.532	0.499	0.501	−0.067
SNP10	154	2.000	1.744	0.618	0.357	0.427	0.428	0.163
SNP11	154	2.000	1.996	0.692	0.513	0.499	0.501	−0.028
SNP12	154	2.000	1.190	0.297	0.162	0.160	0.160	−0.015
SNP13	154	2.000	1.705	0.604	0.442	0.414	0.415	−0.067
Mean	154.000	2.000	1.595	0.515	0.342	0.345	0.346	0.009
SE	0.000	0.000	0.093	0.051	0.043	0.041	0.042	0.023

*n*, number of samples; *Na*, No. of different alleles; *Ne*, No. of effective alleles = 1/(Σp_i_^2^); *I*, Shannon’s information index = −1 × Σ(p_i_ × Ln(p_i_)); *Ho*, observed heterozygosity = No. of Hets/*N*; *He*, expected heterozygosity = 1 − Σp_i_^2^; *uHe*, unbiased expected heterozygosity = (2*N*/(2*N* − 1)) × *He*; *F*, fixation index = (*He* − *Ho*)/*He* = 1 − (*Ho*/*He*); p_i_ is the frequency of the i^th^ allele for the population.

## Data Availability

Data in the form of sequences obtained are available in the Supplementary Materials (File S1). The genotype datasets from hive and flower samples used in this study are publicly available at http://doi.org/10.5281/zenodo.14772790 (accessed on 31 January 2025).

## Supplementary Materials

The following supporting information can be downloaded at https://www.mdpi.com/article/10.3390/genes16030301/s1. Figure S1: Example of two representative samples encompassing all genotyped alleles; Table S1a: SNPs that were monomorphic in our set and therefore were not included in this SNaPshot panel; Table S1b: SNPs with a low frequency (<5%) of the minor allele and therefore were not included in this SNaPshot panel; Table S2: PCR and SNaPshot primers for candidate SNPs used in SNaPshot assay; Table S3a: Composition of the reaction mixture of Multiplex 1; Table S3b: Composition of the reaction mixture of Multiplex 2; Table S3c: Composition of the reaction mixture of Multiplex 3; File S1. Sequence of PCR amplicons containing analyzing SNPs.

## Abbreviations

The following abbreviations are used in this manuscript:

SNP	Single-nucleotide polymorphism
VR	Varroa resistance
VSH	Varroa-specific hygiene
SMR	Suppressed mite reproduction
DBR	Drone-based reduction
WBR	Worker-based reduction
WGS	Whole-genome sequencing
WES	Whole-exome sequencing
GWAS	Genome-wide association study
QTL	Quantitative trait locus
PCR	Polymerase chain reaction
HWE	Hardy–Weinberg equilibrium
DWW	Deformed wing virus
BQCW	Black queen cell virus
SBV	Sacbrood virus
CBPV	Chronic bee paralysis virus
IAPV	Israeli acute paralysis virus
PRR	Pattern recognition receptor
