# Supplementary material for: Genetic Diversity in Candidate Single-Nucleotide Polymorphisms Associated with Resistance in Honeybees in the Czech Republic Using the Novel SNaPshot Genotyping Panel
Source: Genes (Basel). 2025 Mar 1;16(3):301. doi: 10.3390/genes16030301 (PMC11942514; doi:10.3390/genes16030301)
Supplement: Supplementary file 1 [file genes-16-00301-s001.zip › Figure S1.pdf]

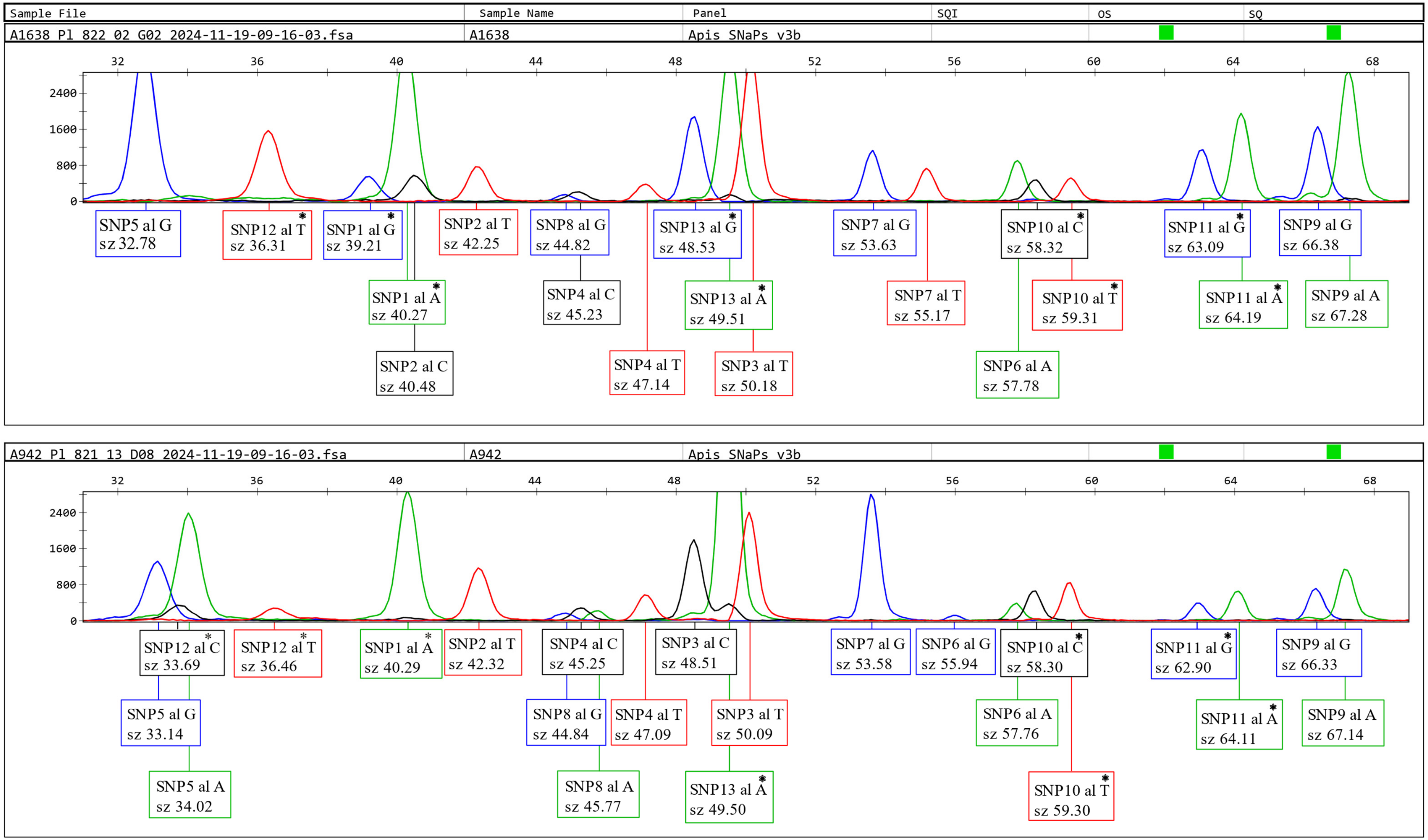

Figure S1: Example of two representative samples encompassing all genotyped alleles. * The reverse primers were used; therefore, the detected alleles are complementary to those determined by SNaPshot.

SNPs genotypes:

Sample	SNP1*	SNP2	SNP3	SNP4	SNP5	SNP6	SNP7	SNP8	SNP9	SNP10*	SNP11*	SNP12*	SNP13*
A1638	CT	CT	TT	CT	GG	AA	GT	GG	GA	GA	CT	AA	CT
A942	TT	TT	CT	CT	GA	GA	GG	GA	GA	GA	CT	GA	TT
