# Supplementary material for: Genetic Diversity in Candidate Single-Nucleotide Polymorphisms Associated with Resistance in Honeybees in the Czech Republic Using the Novel SNaPshot Genotyping Panel
Source: Genes (Basel). 2025 Mar 1;16(3):301. doi: 10.3390/genes16030301 (PMC11942514; doi:10.3390/genes16030301)
Supplement: Supplementary file 1 [file genes-16-00301-s001.zip › File S1.pdf]

File S1. Sequence of PCR amplicons containing analyzing SNPs. SNP position marked red and bold, genotype in the head of sequence

SNP1

>A1095_SNP1_TT
TGAAGCGGTGTGCAGCAGTGGCGTAGTCGTCGATCCGTCCGCGTACAGACGCCCACGGGTTTTTCATTCGGTGTG
AACAAAGAATTCGCCAATATTTTACACTCGATTCGGATCCTGCGACTCGCGAATATCCGTGTTCCTTCCTCTCTT
TCTCACC**T**TCTCTCTTTCCATCTTCCTTTCTCTTTCTCCCGTGGTTCAATCGAACATACGATCCATAT

>A1113_SNP1_CC
TGAAGCGGTGTGCAGCAGTGGCGTAGTCGTCGATCCGTCCGCGTACAGACGCCCACGGGTTTTTCATTCGGTGTG
AACAAAGAATTCGCCAATATTTTACACTCGATTCGGATCCTGCGACTCGCGAATATCCGTGTTCCTTCCTCTCTT
TCTCACC**C**TCTCTCKTTCCATCTTCCTTTCTCTTTCTCCCGTGATTCAATCGAACATACGATCCATAT

SNP2

>A1998_SNP2_TT
TTCCTGGCCACCGATGCCACCCACGTACCTCGAAGCTGCTGTTGCGAACAACGCTCCTCCTTTTTCTTCTTCTTT
TTTACCCATTTTTTCTCCTCTCCACTCCACTTTTCTCAACCTTCTTTCCTTCTTCTT**T**TTCTTCTTCTTCTTTTT
CTGTGCTCCACGAGAGAAAGAGAGAGATTCGTGGAAAGGATCTCGATCTTCAAGAATGGCCGTCGTTTGGAATCG
ACGACAAAAGCGCCCATGGGAGAACAAGGGTTTATCCTATGCCTACGTTAGCCGTATCCGCGATTGGGGGCGTTT
ATGGCGTTTTGTCTCTTT

>A2466_SNP2_CC
TTCCTGGCCACCGATGCCACCCACGTACCTCGAAGCTGCTGTTGCGAACAACGCTCCTCCTTTTTCTTCTTCTTT
TTTACCCATTTTTTCTCCTCTCCACTCCACTTTTCTCAACCTTCTTTCCTTCTT**C**TTCTTCTTCTTTTTCTTTTT
CTGTGCTCCATGAGAGAGAAAGAGATTCGCGAAAAGGATCTCGATCTTCAAGAGTGGCCGTCGTTTGGAATCGAC
GACAAAAGCGCCCATGGGAGAACAAAGGTTTATCCTATGCCTACGTTAGCCGTATCCGCGATTGGGGGCGTTTAT
GGGGTTTTGTCTCTTT

SNP3

>A074_SNP3_TT
CGGAATCAAAAGCAGGATTTACGCCGCTACATTTGAGTGCACAAGAAGGCCATAC**T**GATATGTCAACACTACTTA
TTGAGCACAAAGCAGATACAAATCACAAAGCAAAGGTAATAT

>A447_SNP3_TC(Y)
CGGAATCAAAAGCAGGATTTACGCCGCTACATTTGAGTGCACAAGAAGGCCATAC**Y**GATATGTCAACACTACTTA
TTGAGCACAAAGCAGATACAAATCACAAAGCAAAGGTAATAT

SNP4

>A1621_SNP4_TT
AACGTTGGACCAATTGATGGAACCGTCCGATGTTGTGGGCAACATAAGATTCAGCCATCCAACATTATACGTCTT
CCCCGGTGGCCAAGGTGATGCCGCCCTCTTTGGCATAAACGGTTTTAACATGCTGGTCGACGG**T**GGTTTCGCAAG
GAAAGCCTGCTTTTGGGACTTTACCAGGCATCTCGACCGCCTCGACGCGGTCTTAGTTACTAGAATAAATAATAG
CAATGTCGGCGG

>A3224_SNP4_CC
AACGTTGGACCAATTGATGGAACCGTCCGATGTTGTGGGCAACATAAGATTCAGCCATCCAACATTATACGTCTT
CCCCGGTGGCCAAGGTGATGCCGCCCTCTTTGGCATAAACGGTTTTAACATGCTGGTCGACGG**C**GGTTTCGCAAG
GAAAGCCTGCTTTTGGGACTTTACCAGGCATCTCGACCGCCTCGACGCGGTCTTAGTTACTAGAATAAATAATAG
CAATGTCGGCGG

SNP5

>A1366_SNP5_AA
CGGATTCCAACGTAACGATCGGTGGACAAGAATCTGGGATAAAAAATTTTTCATTCGATCAGAATCAAGAATCTT
TGTTTTCGATCAATGATCAATCGGCCGATAAGATCTTGAACAAGAACGTCGAGGACAATTGGAACGAACGTGAGA
GGCG**A**AGAACGAGCGAATTTCAATTCGGGGCATCGATCGAATCCGATAATTCG

>A3224_SNP5_GG
CGGATTCCAACGTAACGATCGGTGGACAAGAATCTGGGATAAAAAATTTTTCATTCGATCAGAATCAAGAATCTT
TGTTTTCGATCAATGATCAATCGGCCGATAAGATCTTGAACAAGAACGTCGAGGACAATTGGAACGAACGTGAGA
GGCG**G**AGAACGAGCGAATTTCAATTCGGGGCATCGATCGAATCCGATAGTTCG

SNP6

>A1206_SNP6_AA
CCTGCGCGACTGACTAGACATTATCGTACGCATACTGGTGAAAAACCGTATCAATGCGAATATTGTAGTAAATCG
TTTTCCGTAAAAGAGAATTTAAGCGTTCATCGTCGTATACATACAAAAGAACGGCCTTATAAGTGTGACGTGTGC
GAACGCGCGTTTGAACATAGTGGTAAACTACATCGACATATGCGAATTCATACCGGCGAACG**A**CCGCATAAGTGT
ACTGTCTGTTCAAAAACGTTCATTCAAAGCGGACAATTAGTAATTCATATGCGCACGCATACCGGAGAAAAACCA
TACGTTTGCAAAGCATGTGGCAAGGGATTCACTTGCTCAAAGCAACTGAAGGTACATACGCGTACGCATACCGGG
GAAAAACCA

>A1221_SNP6_GG
CCTGCGCGACTGACTAGACATTATCGTACGCATACTGGTGAAAAACCGTATCAATGCGAATATTGTAGTAAATCG
TTTTCCGTAAAAGAGAATTTAAGCGTTCATCGTCGTATACATACAAAAGAACGGCCTTATAAGTGTGACGTGTGC
GAACGCGCGTTTGAACATAGTGGTAAACTACATCGACATATGCGAATTCATACCGGCGAACG**G**CCGCATAAGTGT
ACTGTCTGTTCAAAAACGTTCATTCAAAGCGGACAATTAGTAATTCATATGCGCACGCATACCGGAGAAAAACCA
TACGTTTGCAAAGCATGTGGCAAGGGATTCACTTGCTCAAAGCAACTGAAGGTACATACGCGTACGCATACCGGG
GAGAAACCA

SNP7

>A1621_SNP7_TT
AGGCATCAGGCGTTCGAGGCTGAGATAGCGAGCAACAAGGATCGCTTGCAGCAGTTGCAACAGGCGGCCGAGGAG
TTGATCCAACAGAAGCCAGACCTGGCCGAGATCATCAAGCCGAAGGTGGCCGAATTGGCCGATCAATTCGAGGAG
CTCGAAACGACCACGCACGACAAAGGGGAACGATTGTTCGACGCGAATCGGGAGGT**T**CTCATCCACCAGACCTGC
GACGACATCGACTCGTGGATGAACGAGTTGGAGAAGCAAATCGAGAGCACGGACACCGGGTCAGATCTGGCCTCG
GTCAACATTCTGATGCAGAAGCAACAGATGATCGAGACGCAGATGGCGGTGAAGGCGAGGCAGGTTACC

>A2475_SNP7_GG
AGGCATCAGGCGTTCGAGGCTGAGATAGCGAGCAACAAGGATCGCTTGCAGCAGTTGCAACAGGCGGCCGAGGAG
TTGATCCAACAGAAGCCAGACCTGGCCGAGATCATCAAGCCGAAGGTGGCCGAATTGGCCGATCAATTCGAGGAG
CTCGAAACGACCACGCACGACAAAGGGGAACGGTTGTTCGACGCGAATCGGGAGGT**G**CTGATCCACCAGACCTGC
GACGACATCGACTCGTGGATGAACGAGTTGGAGAAGCAAATCGAGAGCACGGACACGGGGTCAGATCTGGCCTCG
GTCAACATTCTGATGCAGAAGCAACAGATGATCGAGACGCAGATGGCGGTGAAGGCGAGGCAGGTTACC

SNP8

>A2466_SNP8_AA
TTATCCTCCCCTCGAGCGATAAAATTTCTTCTTTCCTTATCGTAGATAACGTTCATACTGACGGCG**A**TGACGCTG
GTCGCCTCGAGCACCGATACCGCGGATCTACCGAGGCCGAGCTACGCCAGGACGATGCTGAAGGTGTCGCGAAGC
CTTCCGAGCGAGACCGAAGAGT

>A2649_SNP8_GG
TTATCCTCCCCTCGAGCGATAAAATTTCTTCTTTCCTTATCGTAGATAACGTTCATACTGACGGCG**G**TGACGCTG
GTCGCCTCGAGCACCGATACCGCGGATCTACCGAGGCCGAGCTACGCCAGGACGATGCTGAAGGTGTCGCGAAGC
CTTCCGAGCGAGACCGAAGAGT

SNP9

>A1095_SNP9_GG
AAGTTCCCGTCAGAGTCGGATTTATCCAGCCTAGAAGC**G**TCGTTAGAGAACGAAGGATCGTTGAGAAAAATAGTG
AAAAGACTGGCACCTGAGAAGAATGGCGAGTACCAAATTTATCAAGGTTCCTAACCAATAATCTTATACTAACTC
CTAAATGTACCGCAATTTACACTACTCTCTTCTCGTTCCATCTCTAAAGTAC

>A1113_SNP9_AA
AAGTTCCCGTCAGAGTCGGATTTATCCAGCCTAGAAGC**A**TCGTTAGAGAACGAAGGATCGTTGAGAAAAATAGTG
AAAAGACTGGCACCTGAGAAGAATGGCGAGTACCAAATTTATCAAGGTTCCTAACCAATAATCTTATACTAACTC
CTAAATGTACCGCAATTTACACTACTCTCTTCTCGTTCCATCTCTAAAGTAC

SNP10

>A1233_SNP10_AA
TTGGCGAATGGGAAACAGGAAAAGGTGGAGAGTATATATCGAAAAATGGC**A**CGTATAAACGGGCTTCAAATCTCG
GAAGAGGCGATCGGAGCGTTCAAAGATTTGAACATGGTGAAGACTGAAAAGGTACGAGGAATCGACAATTTTTAT
TATATTTTAAATGAAATCGAATTAAATTAATTACGCCTAACTAAATTTATCTTATTTTTCAGACGGATCAAATAG
TGATGAAAAGTGACGAAAAGAAATCA

>A3314_SNP10_GG
TTGGCGAATTGGAAACAGGAAAAGGTGGAGAGTATATATCGAAAAATGGC**G**CGTATAAACGGGCTTCAAATCTCG
GAAGAGGCGATCGGAGCGTTCAAAGATTTGAACATGGTGAAGACTGAAAAGGTACGAGGAATCGACAATTTTTAT
TATATTTTAAATGAAATCGAATTAAATTAATTACGCCTAACTAAATTTATCTTATTTTTCAGACGGATCAAATAG
TGATGAAAAGTGACGAAAAGAAATCA

SNP11

>A1206_SNP11_TT
CAAAACATCTGTAAACGCCTCGAATCCGCCTTTGAAATTGGAACGAATCGAGGAAAAGACTTGTTTACAGATGTC
TACAAAGGGACTAACTATAGCAAAAAATTTAGATTACGAGGATATCGAATCGCT**T**CCGGAAATTATATCACCACC
TCCTAAAACACCTCCTGCCTTGCCYCCGAAACCRCAGTTTAAAAAYAACACGTTGATCTTGAAAAAAATTCCTAG
TCCTTCGTTTCTGATCGATGAGACACGAAAGAAACAACAACTTCTTGAATCCGG

>A1221_SNP11_CC
CAAAACATCCGTAAACGCCTCGAATCCGCCTTTGAAATTGGAACGAATCGAGGAAAAGACTTGTTTACAGATGTC
TACAAAGGGACTAACTATAGCAAAAAATTTAGATTACGAGGATATCGAATCGCT**C**CCGGAAATTATATCACCACC
TCCTAAAACACCTCCTGCCTTGCCTCCGAAACCACAGTTTAAAAATAACACGTTGATCTTGAAAAAAATTCCTAG
TCCTTCGTTTCTGATCGATGAGACACGAAAGAAACAACAACTTCTTGAATCCGG

SNP12

>A1621_SNP12_AA
AAAAACATCCTCTAATCTATATCATGTAATGATACATAATACATGAATAAGAGTGGAAATTATASTTACTAAATG
AAATAAATTTATCATCTTACTAGTGTGTTCAAAAAATAGTGCAGAA**A**TGGAGAATGACACCGCTCCAACTCTCCA
GTCATCTTTGGGTATTGGAGAATCGTATTTAGAAGTTCGGGGGATTGCAGCGGGTAGTATAGACATGCGGGAATT
CATCAACGAGTCCTCGTCTTCATATTCGAAATCTTCCATTCCCATTGCCAACAGATTCCTACCAGARATTCGCAT

>A2475_SNP12_GA(R)
AAAAACATCCTCTAATCTAYATCATSTAATGATACATAATACATGAATAAGAGTGGATTTTATACTTACTAAATG
AAATAAATTTATCATCTTACTAGTGTGTTCAAAAAATAGTGCAGAA**R**TGGAGAATGACACCGCTCCAMCTCTCCA
GTCATCTTTGGGTATTGGAGAATCGTATTTAGAAGTTCGGGGGATTGCAGCGGGTAGTATAGACATGCGGGAATT
CATCAACGAGTCCTCGTCTTCATATTCGAAATCTTCCATTCCCATTGCCAACAGATTCCTACCAGAGATTCGCAT

SNP13

>A1560_SNP13_TT
CAAGCTGCGATTCAATCTGGCGAATATGACCATACGAAAAATTATCCCTTCGATGTCGATCAATGGCGTGGTAAA
ATATTTGTATTTTAATTAATATTGTATTTTGCTTCTTGAATCACTTAATATTC**T**ATTCACTTAATATTCAATTTC
GTTACTTGAATTTCTCAATTTTTAAATTGTTAGAATATATTCCACATTCTATTTCACGTTACTTGTTCAAGGTAT
GACTTTTGTAACCGTACCAAGATACAAAGGTGTACCTTCTTCTTTGAACGTGATATCTGAGAAAATTGGCAACGG
TGGACGACTTCTACAACCGTATCCTGATTGGTCGTGGG

>A1893_SNP13_CC
CAAGCTGCGATTCAATCTGGCGAATATGACCATACGAAAAATTATCCCTTCGATGTCGATCAATGGCGTGGTAAA
ATATTCGTATTTTAATTAATATTGTATTTTGCTTCTCGAATCACTTAATATTC**C**ATTCACTTAATATTCAATTTC
GTTACTTGAATTTCTCAATTTTTAAATTGTTAGAATATATTCCACATTYTATTTCACGTTAYTTGTTCAAGGTAT
GACTTTTGTAACCGTACCAAGATACAAAGGTGTACCTTCTTCTTTGAACGTGATATCTAAGAAAATTGGCAACGG
TGGACGACTTCTACAACCGTATCCTGATTGGTCGTGGG
