# Supplementary material for: Genetic Diversity in Candidate Single-Nucleotide Polymorphisms Associated with Resistance in Honeybees in the Czech Republic Using the Novel SNaPshot Genotyping Panel
Source: Genes (Basel). 2025 Mar 1;16(3):301. doi: 10.3390/genes16030301 (PMC11942514; doi:10.3390/genes16030301)
Supplement: Supplementary file 1 [file genes-16-00301-s001.zip › Table S1a,b.pdf]

Table S1a. SNPs that were monomorphic in our set, and therefore were not included in this SNaPshot panel.

Gene name, symbol *	SNP	Positive allele	Type	Associated trait	Location (Amel_HAv3.1)	PCR primer F (5′→ 3′)	PCR primer R (5′→ 3′)	Source
GB17328, GB50048, *Ecdysone-induced protein 93F (Eip93F/ Mblk-1)*	A/G	A	Non-synonymous (Asn/Thr,)	SMR	LG15:7011360	CGGCGCAGATGAGCAAAGT	GGGATTTGTTGCTGCGAGCT	[43]
GB17328, GB50048, *Ecdysone-induced protein 93F (Eip93F/ Mblk-1)*	A/G	A	Non-synonymous (Gln/Arg)	SMR	LG15:7011549	TTTCACGCCGTTCAACTTTTG	TCGCCCTCTGGTCCCTTG	[43]
GB17328, GB50048, *Ecdysone-induced protein 93F (Eip93F/ Mblk-1)*	T/C	C	Non-synonymous (Leu/Pro)	SMR	LG15:7021778	AGAAGCGCCGAGTTGTGGA	TGATGCGACCAAGACCAAGTTT	[43]
GB50526, *sodium-coupled monocarboxylate transporter 1 (LOC410626)*	G/A	A	Synonymous	SMR	LG15:4695209	TCTCAACACGGCCAATAACTCG	GCCCCGTTCTGCTCGTTCT	[42]
GB48134, *L-lactate dehydrogenase (LOC411188)*	C/T	T	Synonymous	VSH	LG7:13958516	ATGGCATCGTTGAGAAGTAAACTG T	TCGGACTGTATTTCACCAACTCG	[44]
GB53150, *scribble planar cell polarity protein (scrib/lap4)*	T/C	C	Synonymous	VSH	LG4:3367093	ACCCAAGCCTATCACCAACGA	TCATTTCATCTAGCTACCAAGCAC A	[44]
GB42579, *1-phosphatidylinositol 4,5-bisphosphate phosphodiesterase (LOC408996 /norpA2)*	G/A	A	Intron	VSH	LG9:10146906	GGCAAAGCGGACACGATCA	TAATGCGCGAGCGGTTGA	[44]
GB15698, GB55405, eyegone/Eyg, SNP labeled as AMB-00573174	G/A	A	Exon/5′UTR	VSH	LG2: 14143654	CGCCTTAAATCTTGATCTCACCTG	GCGCACTCGTTTGACAGCTC	[50]

GB47452, *dynein heavy chain, cytoplasmic (Dhc64C)*	A/G	G	Synonymous	VR	LG1:14759170	GCAATGATGGCACAATTAAAAGAA	TTCCCAAGTCAATGCTTCTTCTTC	[44]
GB53340, *Spectrin beta chain isoform X1 (beta-Spec)*	G/T	T	Non-synonymous (Gln/Lys)	VR	LG9:11462544	AGCCGAGAGTTGCACAAATTCTT	GAGGCTTCTCCGAGGTGTTCA	[44]
GB55322, homeobox protein engrailed-1a (LOC725151/En)	A/G	G	Non-synonymous (Thr/Ala)	VR	LG1:17798420	CTCGCCAGGATCAGCAACAG	GGGGCCTTGTCGGAGATAATC	[44]
GB15987, GB45774, Dscam family member AbsCAM (Abscam/Dscam2)	T/C	C	Synonymous	VR	LG6:1629786	CAGCGTTTTACTGATTAGGGTCTTT T	CCGCTTGATTGCTACCAGCTAC	[44]
GB41850, *Hemolectin/hemocytin (LOC411597/Hml)*	T/C	Not assesed	Non-synonymous (Glu/Lys)	Immune response	LG8:7315123	GACCTTCAACAGCACCTCCAA	TGTCAAATAAAATTTGCGATACTG G	[19]
GB10346, GB40100, partitioning defective 3 homolog (LOC726759)	G/A	A	Synonymous	Immune response	LG13:6618371	GGATGCAATGGAAACACTGAGAA	CGCTGGCTTTCGTGGAATC	[44]

* Gene and protein designation according to NCBI and Uniprot databases; VR: varroa resistance; VSH: Varroa specific hygiene; SMR: Suppressed mite reproduction

Table S1b. SNPs with a low frequency (<5 %) of the minor allele and therefore were not included in this SNaPshot panel.

Gene name, symbol *	SNP	Positive allele	Type	Associated trait	Location (Amel_HAv3.1)	PCR primer F (5′→ 3′)	PCR primer R (5′→ 3′)	Source
GB51513, UPF0183 protein CG7083 (LOC409639), SNP labeled as AMB-00913945	A/G	G	Synonymous	VSH	LG3: 4846557	ATGGGTGCTGGAGGTGGAAG	TTCACAAGTATCCCCAAATAAC ACC	[50]
SNP labeled as AMB-01079196, nearest genes are LOC550886 atlastin and LOC408822	A/G	G	Intergenic	VSH	LG5: 1036781	TCATTGATATGATTGCGAACTTG TT	GGGTTGACTGGAATCGAGAGAG	[50]
GB46431, eukaryotic translation initiation factor 3 subunit A (eIF3a/ eIF3-S10)	G/A	A	Synonymous	VR	LG1:12197225	TTCCGCAACAAAAAATGGACA	GGCCATGCGTGCAATATCA	[44]
GB49171, sidekick cell adhesion molecule (sdk/IGFn3-8)	C/T	Not assesed	Non-synonymous (Thr/Met)	Immune response	LG3:10174579	TGCGGAGATCATATAGGAATTG GT	CGCGAAGAATATGAAAAAAAAT ACG	[19]
GB50508, fibrillin-2/ Eater-like (LOC72442/ NimC1)	T/C	Not assesed	Non-synonymous (Gly/Asp)	Immune response	LG15:5255693	CGCGTACACTCGACTGCAATTA G	TGTGAACGATACGATTGATTACG TG	[19]

* Gene and protein designation according to NCBI and Uniprot databases; VR: varroa resistance; VSH: Varroa specific hygiene; SMR: Suppressed mite reproduction
