# Supplementary material for: Genetic Diversity in Candidate Single-Nucleotide Polymorphisms Associated with Resistance in Honeybees in the Czech Republic Using the Novel SNaPshot Genotyping Panel
Source: Genes (Basel). 2025 Mar 1;16(3):301. doi: 10.3390/genes16030301 (PMC11942514; doi:10.3390/genes16030301)
Supplement: Supplementary file 1 [file genes-16-00301-s001.zip › Table S2.pdf]

Table S2: PCR and SNaPshot primers for candidate SNPs used in SNaPshot assay.

SNP	PCR primer F (5′→ 3′)	PCR primer R (5′→ 3′)	Amplicon length (bp)	SNaPshot primer (5′→ 3′)	SNaPshot primer length	SNaPshot primer orientation (F/R)
SNP1	CGGCAGATACGGTTGGAATG	CACGCTTACACGATACGAAAAAAC	320	*GACTGACTGA*GAAAGGAAGATGGAAAGAGAGA	32	R
SNP2	GCCCTTTTTCCTTGTTCCTTTG	GTCGTCATCCGTTTCAGACAAATC	411	*GACTGACTGA*TCAACCTTCTTTCCTTCTTCTT	32	F
SNP3	CGGAATCAAAAGCAGGATTTACG	AAGCGGCGTGAGACCATTCTATA	202	*GACTGACTGACTGACTGACTGACTG*TGCACAAGAAGGCCATAC	43	F
SNP4	GGCCTGTCAGCAATCACCAG	GCCGCCGACATTGCTATTATT	294	*GACTGACTGACTGACTGACT*TAACATGCTGGTCGACGG	38	F
SNP5	ACGGAGGATCAAACAATCAGAGA[illegible]	AGATCCCGCGATTCTCGTTC	439	*GACT*GAACGAACGTGAGAGGCG	22	F
SNP6	TTGCGCTCACACGGTAAAGAA	CCCGCAAATGTCGCAAGTATA	474	*GACTGACTGACTGACTGACTGACTGACTGACTGAC*AATTCATACCGGCGAACG	53	F
SNP7	CCGCCCAGGACGAGACTTAC	CTCCGCCTGTTTGTCCAACTC	443	*GACTGACTGACTGACTGACTGACTGACTGA*CGACGCGAATCGGGAGGT	48	F
SNP8	ATTCGCGCGTTCATTGGTC	CGCCGATTTCTCCCTACGTT	345	*GACTGACTGACTGACTGACT*ACGTTCATACTGACGGCG	38	F
SNP9	CGAGTTAAATTTAAACTTTTAGTCAAA	GGGAAAGAAAGGCGAAGAAAGTA	277	*GACTGACTGACTGACTGACTGACTGACTGACTGACTGACTGA*GGATTTATCCAGCCTAGAAGC	63	F
SNP10	TTGGCGAATGGGAAACAGG	CAACAGCCGAGTAAGCATCACC	299	*GACTGACTGACTGACTGACTGACTGACTGACTG*ATTTGAAGCCCGTTTATACG	53	R
SNP11	CAAAGATCAAGATGGAAGAAACG[illegible]G	CCGGATTCAAGAAGTTGTTGTTTC	343	*GACTGACTGACTGACTGACTGACTGACTGACTGACTGA*GGTGGTGATATAATTTCCGG	58	R
SNP12	GCAAAATGCAGAAGAAGAGGAAAAA	ATGCGAATCTCTGGTAGGAATCTGT	352	*GACT*GAGCGGTGTCATTCTCCA	22	R
SNP13	TGGCAAATTCGATGAACGTGA	CCCACGACCAATCAGGATACG	409	*GACTGACTGACTGACTG*GTAACGAAATTGAATATTAAGTGAAT	43	R

SNaPshot primers contain specific sequence and GACT tail (italics)
