# Supplementary material for: Genetic Diversity in Candidate Single-Nucleotide Polymorphisms Associated with Resistance in Honeybees in the Czech Republic Using the Novel SNaPshot Genotyping Panel
Source: Genes (Basel). 2025 Mar 1;16(3):301. doi: 10.3390/genes16030301 (PMC11942514; doi:10.3390/genes16030301)
Supplement: Supplementary file 1 [file genes-16-00301-s001.zip › Table S3a-c.pdf]

Table S3a. Composition of the reaction mixture of Multiplex 1.

Components	Volume (µL)
H_2O	3.3
Combi PPP Mix	5.0
SNP2-F	0.1
SNP2-R	0.1
SNP3-F	0.2
SNP3-R	0.2
SNP7-F	0.1
SNP7-R	0.1
SNP4-F	0.1
SNP4-R	0.1
SNP11-F	0.1
SNP11-R	0.1
**Total**	9.5
**DNA**	0.5

Table S3b. Composition of the reaction mixture of Multiplex 2.

Components	Volume (µL)
H_2O	3.7
Combi PPP Mix	5.0
SNP1-F	0.1
SNP1-R	0.1
SNP12-F	0.1
SNP12-R	0.1
SNP5-F	0.1
SNP5-R	0.1
SNP6-F	0.1
SNP6-R	0.1
**Total**	9.5
**DNA**	0.5

Table S3c. Composition of the reaction mixture of Multiplex 3.

Components	Volume (µL)
H_2O	3.7
Combi PPP Mix	5.0
SNP8-F	0.1
SNP8-R	0.1
SNP13-F	0.1
SNP13-R	0.1
SNP9-F	0.1
SNP9-R	0.1
SNP10-F	0.1
SNP10-R	0.1
**Total**	9.5
**DNA**	0.5
